# Nucleoside Diphosphate Kinase Family: Evolutionary Analysis and Protective Role in Mitochondrial ROS Production

**DOI:** 10.3390/plants15081156

**Published:** 2026-04-09

**Authors:** Douglas Jardim-Messeder, Ygor de Souza-Vieira, Thais Felix-Cordeiro, Régis L. Corrêa, Gilberto Sachetto-Martins

**Affiliations:** 1 Programa de Biologia Molecular e Biotecnologia, Instituto de Bioquímica Médica Leopoldo de Meis, Universidade Federal do Rio de Janeiro, 21941-902 Rio de Janeiro, RJ, Brazil; 2 Departamento de Genética, Instituto de Biologia, Universidade Federal do Rio de Janeiro, 21941-902 Rio de Janeiro, RJ, Brazil; ygor.vieira3126@gmail.com (Y.d.S.-V.); thafe01@gmail.com (T.F.-C.); regislcorrea@gmail.com (R.L.C.); 3 Institute for Integrative Systems Biology (I2SysBio), Consejo Superior de Investigaciones Cientificas (CSIC), Universitat de València (UV), 46980 Valencia, Spain

**Keywords:** NDPK, molecular evolution, plant mitochondria, reactive oxygen species, antioxidant defense

## Abstract

Nucleoside diphosphate kinase (NDPK) is a ubiquitous enzyme that maintains cellular nucleotide balance by catalyzing the transfer of phosphate groups between nucleoside diphosphates and triphosphates. Although the evolutionary conservation of NDPK is well established, several aspects of its diversification and functional adaptation remain unclear. The central question of this work is how NDPK evolved across plant species, focusing on the Solanaceae family and how its evolutionary history relates to the diversification of its cellular functions. Phylogenetic and molecular dating analyses showed that the division between NDPK groups 1 and 2 predates the divergence of plants and animals, whereas plant-specific NDPK types (I–IV) originated early in streptophyte evolution. Solanaceae species retain a conserved set of NDPK genes, including a type III isoform with features consistent with mitochondrial targeting. Functional assays in isolated potato tuber mitochondria revealed high NDPK activity in the intermembrane space, sustaining ADP supply to oxidative phosphorylation. Activation of mitochondrial NDPK induced a phosphorylative respiratory state, which partially dissipated the mitochondrial membrane potential and significantly reduced reactive oxygen species (ROS) production. GDP and UDP were preferentially phosphorylated, conferring a stronger antioxidant effect than other nucleotides. Consistently, the mitochondrial isoform StNDPK3 was upregulated during tuber development. Together, our results demonstrate that NDPKs are evolutionarily conserved yet functionally diversified enzymes in plants and identify mitochondrial NDPK as a key modulator of mitochondrial redox homeostasis. By linking nucleotide metabolism to Δψ_m_ control and ROS suppression, this study highlights a previously underappreciated antioxidant mechanism that integrates mitochondrial energy metabolism with developmental and stress-related processes in plants.

## 1. Introduction

Nucleoside diphosphate kinases (NDPK), called non-metastatic (NME) in animals, are highly conserved enzymes found in virtually all organisms [1]. They can utilize both ribo- and deoxyribonucleotides, which we collectively refer to as NTPs, acting as phosphoryl donors to an acceptor nucleoside diphosphate (NDP) [2]. NDPKs function by supplying NTPs for macromolecule synthesis (e.g., nucleic acids, lipids, and polysaccharides), protein elongation, microtubule polymerization, and cell signaling in response to stress [3]. The NDPK/NME family is divided into two main groups (group 1 and group 2), where animals NME1, NME2, NME3, NME4, NME-LV, and NMEGp1 are clustered in group 1, and group 2 contains NME5, NME6, NME7, NME8, and NME9 [4]. NDPK from arabidopsis (*Arabidopsis thaliana*) and rice (*Oryza sativa*) plants has been shown to be divided into four NDPK types (I, II, III, and IV), with types I, II, and III grouped in group 1 and type IV in group 2 [5].

In animals, *NME1* was first described as a prototypic metastasis suppressor gene [6]. Cytosolic NME1 suppresses metastasis by promoting receptor recapture through DYNAMIN2, a GTPase motor required for endocytosis [6]. NME3, an isoform found on the mitochondrial outer membrane [7], is also essential for suppressing cancer metastasis [6], possibly by regulating mitochondrial fusion through its interaction with mitofusin (MFN) [8,9]. NME4, an exclusively mitochondrial NME, interacts with the dynamin-like protein OPA1 in the inner membrane and enhances GTP loading onto OPA1 [7,10]. The combined activities of NME3 with MFN at the outer membrane and NME4 with OPA1 at the inner membrane likely maintain a fused mitochondrial network that helps prevent metastasis [6]. Animal *NME* genes are also linked to cellular differentiation and development, including totipotency and nervous system development in Drosophila [11].

It is clear that NDPKs have many other autonomous roles unrelated to the main function, known as moonlight function. Aside from nucleoside kinase function, NDPKs were reported as protein kinases at histidine residues in mammalian cells [12,13], act in regulatory roles by binding to the DNA and several proteins [14], and create complexes with cytoskeletal elements impacting their functions [6]. Compared to animals, plant NDPKs are less studied; however, their function and versatility are highlighted as similar to those of animals’ homologs.

There is an increasing suggestion that plant NDPKs are multifunctional enzymes and involved in development and stress [3,5,15,16,17]. Subcellular localization is crucial for functional specialization as it determines substrate accessibility and the availability of interaction partners. NDPK1 is generally reported to localize in the cytosol, whereas NDPK2 is found in the chloroplast stroma [5]. Mitochondrial-located NDPKs (mit-NDPK) were first reported in arabidopsis (AtNDPK3) and pea (*Pisum sativum*) (PsNDPK3) [18]. Submitochondrial fractionation studies revealed that pea NDPK3 is present both in a soluble form within the intermembrane space (IMS) and tightly bound to the inner mitochondrial membrane [18]. In arabidopsis, NDPK3 displays dual targeting, localizing predominantly in mitochondria but also to the thylakoid lumen of chloroplasts [19,20]. Type IV NDPKs in plants were identified later, and their functions and cellular localization remain poorly defined. The presence of an ER retention signal suggests localization to the endoplasmic reticulum, and some studies detected NDPK activity in the ER and nucleosomes, which may correspond to type IV NDPK; however, this information still needs to be confirmed [5].

Insights into NDPK function were obtained through transgenic manipulation in plants. In potato (*Solanum tuberosum*) roots, overexpression or repression of *NDPK1* revealed its critical role in balancing cellular nucleotide pools and carbohydrate metabolism [21]. Repression of *NDPK1* reduced UTP synthesis but enhanced ATP availability, favoring starch accumulation, whereas overexpression increased UTP production, supporting UDP–glucose formation and cellulose biosynthesis [21]. In potato, *NDPK1* overexpression further stimulated root growth, glycolysis, and respiration while also influencing reactive oxygen species (ROS) dynamics and redox regulation of starch biosynthetic enzymes such as AGPase [22].

In arabidopsis, NDPK2 serves as a central regulator of both oxidative stress responses and phytochrome-mediated light signaling. Expression of NDPK2 is strongly induced by H_2_O_2_ stress, and loss-of-function mutants accumulate higher levels of ROS compared to wild type [23]. In addition, NDPK2 interacts with phytochrome-A and *ndpk2* mutants exhibit partial defects in red- and far-red light responses, including impaired cotyledon opening and greening, indicating its role as a positive component of phytochrome signaling [24,25]. Arabidopsis overexpressing NDPK2 display reduced ROS levels and enhanced tolerance to abiotic stresses, such as cold, salinity, and methyl viologen treatment, establishing this isoform as a key mediator linking ROS homeostasis with environmental signaling [23]. In arabidopsis, functional studies indicate that NDPK3 positively regulates antioxidant enzyme activity under oxidative stress, with overexpressing lines showing enhanced responses to environmental pollutants, while knockouts display reduced antioxidant activity [26]. Collectively, these studies highlight NDPK as a key regulator linking nucleotide metabolism with carbon allocation, energy balance, and redox homeostasis.

The mitochondrial production of ROS is highly dependent on mitochondrial membrane potential (∆ψ_m_). In this context, the activity of mitochondria-associated kinases that preferentially use ATP as a phosphate donor, by promoting an ADP recycling mechanism, provides a constant ADP supply to oxidative phosphorylation leading to ATP synthesis; this is coupled to a decrease in ∆ψ_m_ and consequently promotes a preventive antioxidant role. Indeed, it has been demonstrated that the activities of mt-ADK in plant mitochondria can enable the rate of phosphorylation of NDPs at rates equal to the maximum rate of oxidative phosphorylation [27]. Thus, mt-NDPK activity appears to be capable of promoting an ADP cycling mechanism in IMS from the ATP generated in the mitochondrial matrix.

In the present work, we identified and characterized the NDPK family members in Solanaceae species. The evolutionary analyses demonstrate that the divergence between NDPK groups I and II is highly ancestral, having occurred early in eukaryotic evolution, prior to the emergence of both animals and plants. While NDPK group I (types I, II, and III) was retained throughout Streptophyta, NDPK group II (type IV) appears to have been exclusively retained in tracheophytes. Later, lineage-specific duplication events contributed to the expansion of NDPK paralogs. In addition, we demonstrate that mt-NDPK participates in the control of mitochondrial ROS production by inducing the mitochondrial phosphorylative state, which reduces the ∆ψm and consequently promotes a preventive antioxidant defense. We also observed a differential affinity of potato tuber mitochondria for phosphorylating different NDPs (CDP, GDP, TDP, and UDP), indicating that each nucleotide displays a specific antioxidant potential.

Taken together, these findings highlight NDPKs as evolutionarily conserved functionally versatile enzymes that integrate nucleotide metabolism with cellular signaling, organelle function, and stress responses. Despite substantial advances in animal systems, important gaps remain regarding the evolutionary history, diversification, and mitochondrial functions of NDPKs in plants, particularly in major crop lineages such as Solanaceae. Here, we provide a comprehensive molecular and evolutionary characterization of the NDPK family in Solanaceae and investigate the role of mitochondrial NDPK in regulating mitochondrial redox homeostasis. By linking evolutionary diversification with functional specialization and antioxidant control, this work advances our understanding of NDPK roles in plant energy metabolism and stress adaptation.

## 2. Results

### 2.1. Identification and Phylogenetic Analysis of NDPK Genes

**Figure 1:**
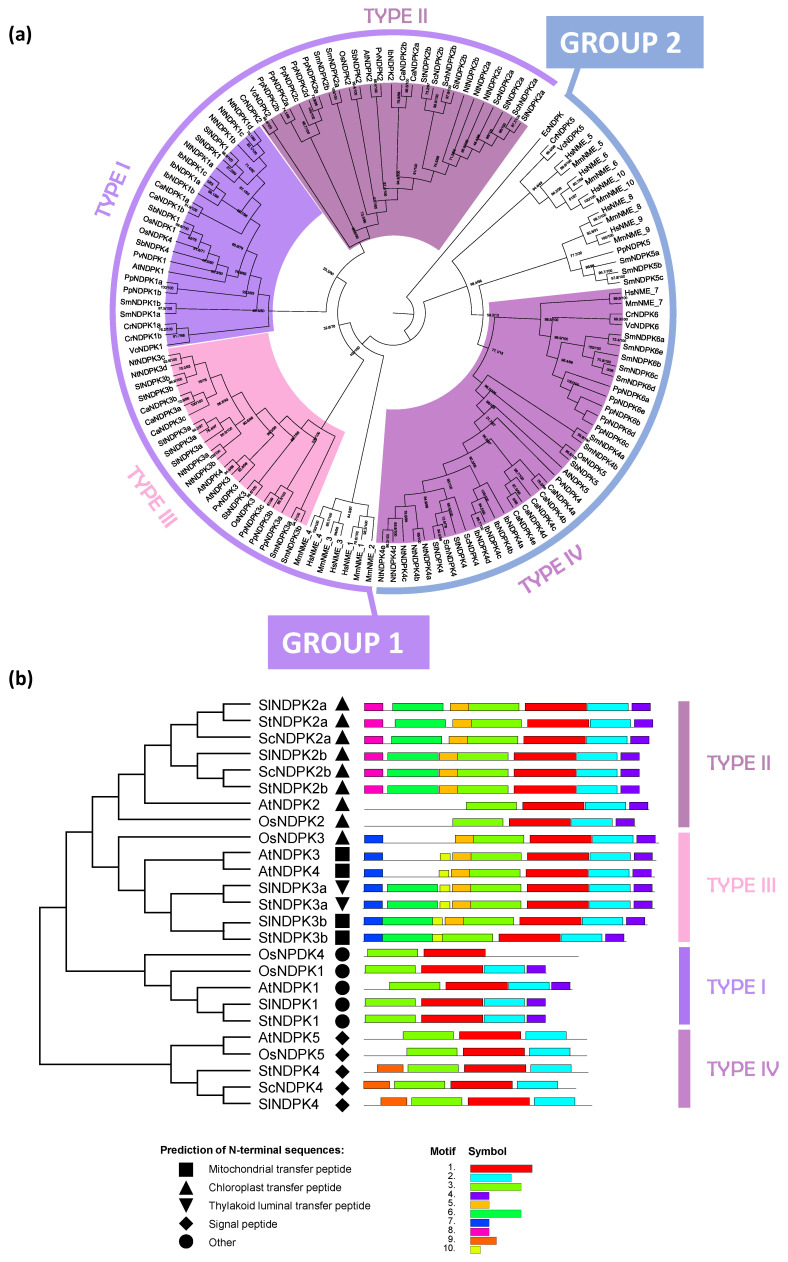
Phylogenetic analysis of NDPK family and conserved motifs. (**a**) Maximum likelihood phylogenetic tree of the NDPK gene family transformed branches to cladogram. The protein sequences were aligned using MUSCLE in MEGA12, and phylogenetic reconstruction was done using the maximum likelihood method under the best model selection in iQtree software (version 1.4.4), with 1000 replicates of bootstrap and approximate likelihood ratio test (ALRt) statistics. Bootstrap values ≥ 70% are indicated at the nodes, while values below this threshold are omitted to improve figure clarity. Species used in the phylogenetic analysis: *A. thaliana* (At), *S. tuberosum* (St), *S. lycopersicum* (Sl), *S. commersoni* (Sc), *S. chilense* (Sch), *N. tabacum* (Nt), *I. batatas* (Ib), *C. arábica* (Ca), *P. vulgaris* (Pv), *O. sativa* (Os), *S. bicolor* (Sb), *P. patens (*Pp), *S. moellendorffii* (Sm), *C. reinhardtii* (Cr), *V. carteri* (Vc), *M. musculus* (Mm), *H*. *sapiens* (Hs), and *E. coli* (Ec). (**b**) Conserved protein motifs in *O. sativa*, *A. thaliana*, *S. tuberosum*, *S. lycopersicum*, and *S. commersoni* sequences. The available sequences for *ScNDPK1*, *ScNDPK3a*, and *ScNDPK3b* are incomplete and were therefore excluded from this analysis. All 10 conserved protein motifs are indicated by colored boxes, and the lines represent non-conserved sequences. The phylogenetic relationships among the analyzed proteins were reconstructed using the maximum likelihood method under the best model selection in MEGA 12. The presence of N-terminal sequences is indicated by symbols, as described in the figure legend.

The NDPK family was analyzed using arabidopsis and rice protein sequences [5] as queries against the genomes of *V. carteri*, *C. reinhardtii*, *S. moellendorffii*, *P. patens*, *I. batatas*, *S. lycopersicum*, *S. tuberosum*, *S. commersonii*, *S. chilense*, *N. tabacum*, *C. arabica*, *P. vulgaris*, and *S. bicolor*. To ensure a robust evolutionary framework and accurately infer the timing and nature of gene duplication events—including lineage-specific duplications—representative species from distinct plant lineages were included. Phylogenetic relationships were inferred using full-length protein sequences from these species, alongside arabidopsis and rice NDPKs, as well as mouse and human NMEs, allowing broader phylogenetic context and improved resolution of ancestral versus lineage-specific events. The tree was rooted with the *Escherichia coli* NDPK sequence, and the proteins from all examined species were consistently included in the reconstruction (Figure 1a).

NDPK genes are present in all known organisms, and their duplications trace back to very early evolutionary events. Based on the phylogeny inferred in the present study, the duplication that gave rise to group 1 and group 2 predates the divergence of animals and plants. In contrast, the divergence among types I, II, and III can be traced back to the origin of Streptophyta. These patterns are further detailed in Section 2.4.

Consistent with previous reports for arabidopsis and rice, plant NDPKs were classified into four types (I–IV) (Figure 1a,b), which fall into two major groups shared with animal NMEs (Figure 1a) [5]. Types I–III belong to group I, which contains functionally characterized plant NDPKs. Type I NDPKs were found in all analyzed species, except for wild potato and wild tomato, possibly because of a badly annotated genome. Lineage-specific gene duplications were evident in tobacco (*NtNDPK1a*, *NtNDPK1b*, *NtNDPK1c*, *NtNDPK1d*), sweet potato (*IbNDPK1a*, *IbNDPK1b*, *IbNDPK1c*), and coffee (*CaNDPK1a*, *CaNDPK1b*) (Figure 1a). Additional duplications were observed in sorghum (*SbNDPK1*, *SbNDPK4*), reflecting the duplication pattern previously reported in rice (*OsNDPK1*, *OsNDPK4*) [5]. Outside angiosperms, distinct clades were identified for *P. patens* (*PpNDPK1a*, *PpNDPK1b*), *S. moellendorffii* (*SmNDPK1a*, *SmNDPK1b*), and the green algae *C. reinhardtii* (*CrNDPK1a*, *CrNDPK1b*) and *V. carteri* (*VcNDPK1*) (Figure 1a).

The type II clade contained at least one gene from all species analyzed (Figure 1a). Gene duplications were identified within the Solanaceae lineage for NDPK2 (*StNDPK2a* and *StNDPK2b*; *SlNDPK2a* and *SlNDPK2b*; *SchNDPK2a* and *SchNDPK2b*; *ScNDPK2a* and *ScNDPK2b*; *NtNDPK2a* and *NtNDPK2b*) as well as independently in coffee (*CaNDPK2a* and *CaNDPK2b)* and tobacco (*NtNDPK2a* and *NtNDPK2c*) (Figure 1a). Outside the eudicots, type II members were also found in Poaceae (*OsNPDK2* and *SbNPDK2*), *S. moellendorfii* (*SmNDPK2a* and *SmNDPK2b*), *P. patens* (*PpNDPK2a*, *PpNDPK2b*, *PpNDPK2c*, *PpNDPK2d*, and *PpNDPK2e*), and the green algae *C. reinhardtii* (*CrNDPK2*) and *V. carteri* (*VcNDPK2*) (Figure 1a).

Type III plant NDPKs were present in nearly all land plants analyzed in the present work, except for wild potato and wild tomato, possibly because of a badly annotated genome. Lineage-specific duplications were observed in coffee (*CaNDPK3a*, *CaNDPK3a*, and *CaNDPK3c*) and tobacco (*NtNDPK3a* and *NtNDPK3b*; *NtNDPK3c* and *NtNDPK3d*) (Figure 1a). Solanaceae lineage-specific duplication was also observed (*StNDPK3a*, *SlNDPK3a*, *NtNDPK3a*, and *NtNDPK3b*; *StNDPK3b* and *SlNDPK3b*) (Figure 1a). Beyond the eudicots, type III members were found in Poaceae (*OsNDPK3* and *SbNDPK3*), *P. pate*ns (*PpNDPK3a*, *PpNDPK3b*, and *PpNDPK3c*), and *S. moellendorfii* (*SmNDPK3a* and *SmNDPK3b*) (Figure 1a).

Type IV represented the only plant-specific NDPK clade within group II (Figure 1a). In this clade, specific duplications were observed in some species, such as *S. moellendorffii* (*SmNDPK4a* and *SmNDPK4b*), coffee (*CaNDPK4a*, *CaNDPK4b*, *CaNDPK4c*, *CaNDPK4d*, and *CaNDPK4e*), sweet potato (*IbNDPK4a*, *IbNDPK4b*, *IbNDPK4c*, and *IpNDPK4d*), and tobacco (*NtNDPK4a*, *NtNDPK4b*, *NtNDPK4c*, *NtNDPK4d*, and *NtNDPK4e*). In addition, within group II, other NDPK clades were present that were likely generated by ancestral duplication events and include sequences from both plants and animals. However, in these clades, NDPK paralogs are retained only in mosses and algae. This pattern may reflect extensive gene loss in other plant lineages or, alternatively, independent retention or acquisition events in these taxa and animals. A broader phylogenetic analysis incorporating additional basal plant lineages, gymnosperms, and early-diverging angiosperms will be essential to resolve these evolutionary relationships.

### 2.2. Protein Motifs and Structural Analysis of Regulatory Residues Related to NDPK Activity

**Figure 2:**
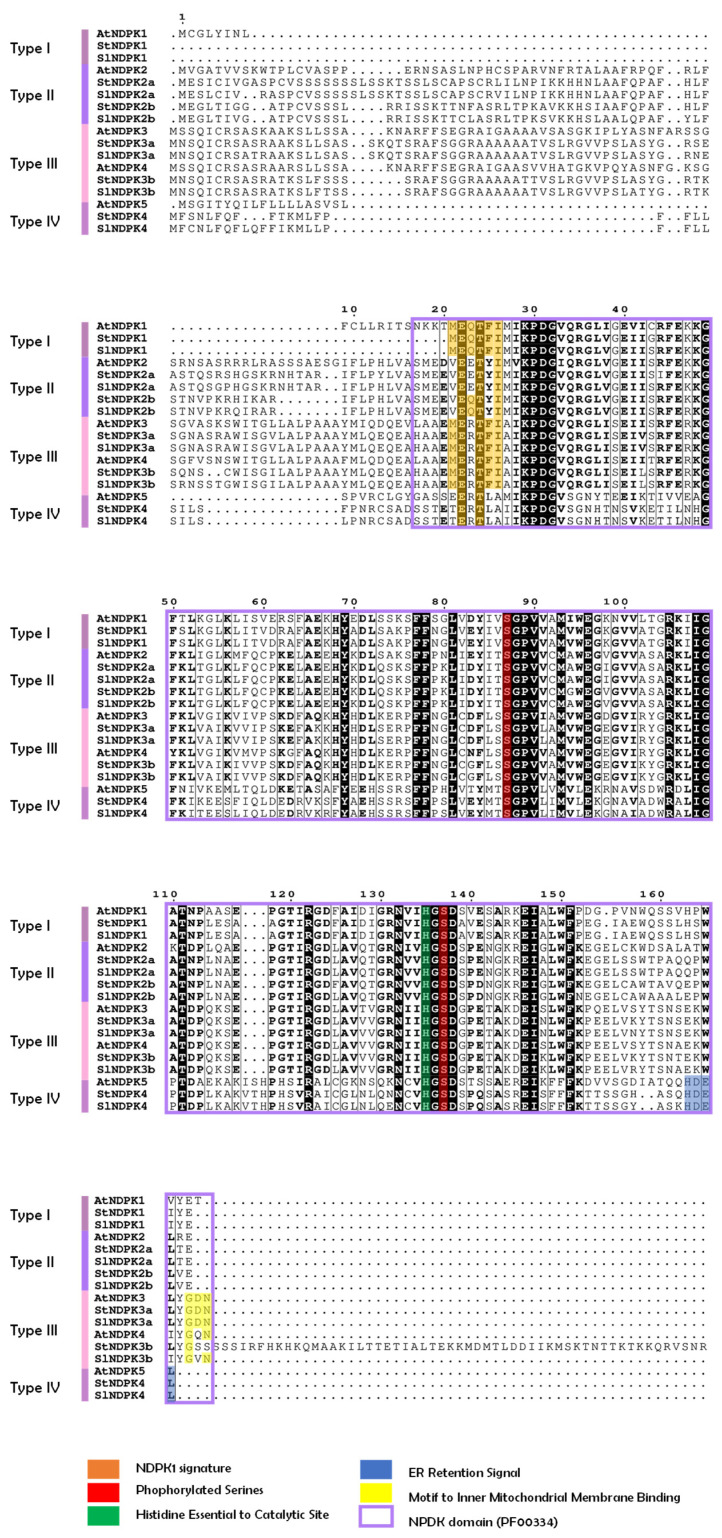
Amino acid sequence analysis of NDPK family in *S. tuberosum* (St), *S. lycopersicum* (Sl), and *A. thaliana* (At). Deduced amino acid sequences of NDPK genes were aligned by Clustal Omega tool in MEGA 12 software. Conserved amino acids are labeled in black and conserved substitutions in bold format. NDPK1 signature, phosphorylated serines, histidine essential to catalytic site, ER retention signal, motif to inner mitochondrial membrane binding, and NPDK domain are indicated by different colors, as indicated in the figure.

To analyze the structural features of plant NDPK proteins, the primary structure of proteins was analyzed in comparison with potato, tomato, arabidopsis, and rice (Figure 1b and Figure 2). A great degree of conservation was observed among NDPK protein motifs among all plant NDPK types (types I to IV) (Figure 1b). Motifs 1, 2, and 3 represented the region of the NDPK domain and seemed to be conserved among all plant NDPK groups (Figure 1b). Indeed, the NDPK domain sequence was conserved among NDPK subtypes, including the serine residues that should be autophosphorylated for activity [28], the histidine residue that was reported as essential for catalytic activity [29], and the catalytic site (Figure 2). The type I NDPK should have a signature “MEQTFI” in the N-terminus [5], and it was conserved among all analyzed species. Additionally, the MEME motif 7 (Figure 1b) may correspond to an organellar targeting signal. Protein alignment analysis revealed an N-terminal feature characteristic of mitochondrial targeting sequences in type III NDPK. Indeed, the motif described as necessary to bind the inner mitochondrial membrane, “GDN” [29] was present in all type III NDPK sequences in the C-terminal region (Figure 2). AtNDPK3, StNDPK3a, and SlNDPK3a proteins retained the conserved “GDN” motif; however, other type III NDPKs exhibited substitutions at this site, with aspartate (D) replaced by glutamine (Q) in arabidopsis Arabidopsis and by valine (V) in potato and tomato. This may indicate that these proteins could not bind to the inner mitochondrial membrane.

Type IV NDPKs are the least studied ones, and their specific characteristics remain unknown. Their primary sequence contains a conserved endoplasmic reticulum (ER) retention signal, consistent with what has been previously reported for NDPK5 in arabidopsis and rice [5], and this feature was conserved across all analyzed species (Figure 2). Notably, proteins with NDPK characteristics were identified in ER microsomal fractions of multiple plant species [5].

### 2.3. Structural Organization of NDPK Genes

**Figure 3:**
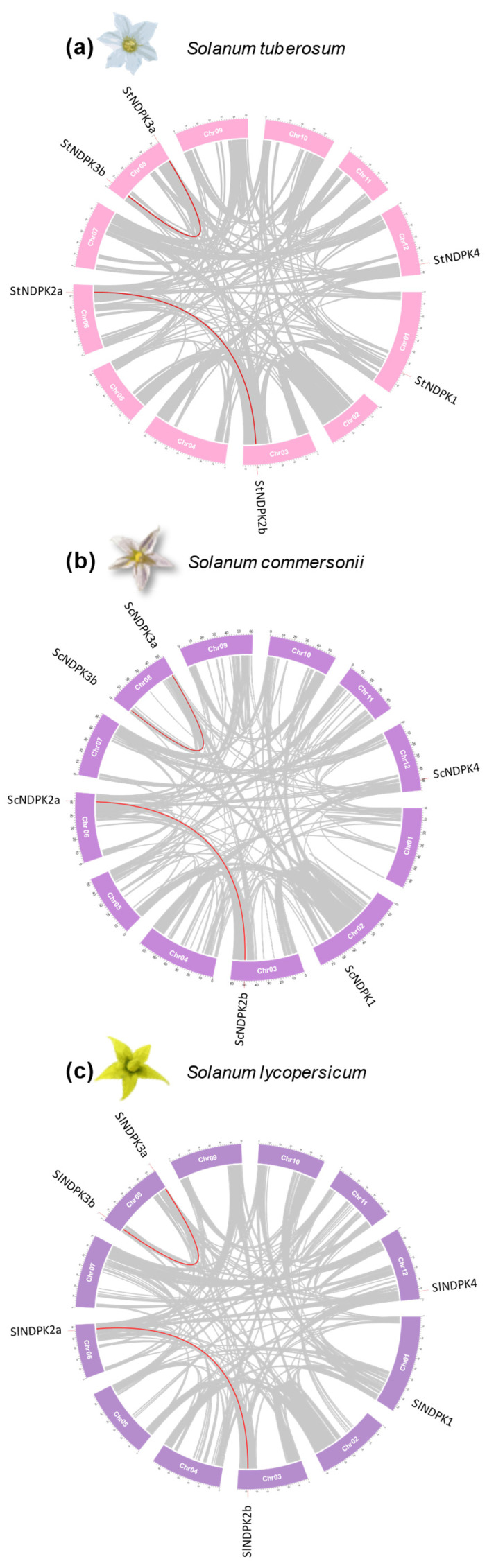
Chromosomal positions and inter-chromosomal groups of duplicated *NDPK* gene pairs in *S. tuberosum* (**a**), *S. commersonii* (**b**), and *S. lycopersicum* (**c**). Gray lines in the background demonstrate all syntenic blocks and the red lines exhibit the segmental or tandem duplication network zones.

To investigate the chromosomal distribution and duplication events within the NDPK family, the genomic positions of each gene in potato, wild potato, and tomato were determined using data obtained from their respective genome databases at NCBI. The genomic positions of all *NDPK* genes are shown in Figure 3. Our analysis of gene duplication revealed that the duplication of the *NDPK2* and *NDPK3* genes was conserved across the examined Solanaceae species. This finding is consistent with the phylogeny (Figure 1). In addition, gene location and the presence of conserved gene blocks between regions suggested that NDPK2 and NDPK3 genes may have been duplicated in a Solanaceae ancestor by whole-genome duplication events (Figure 3).

**Figure 4:**
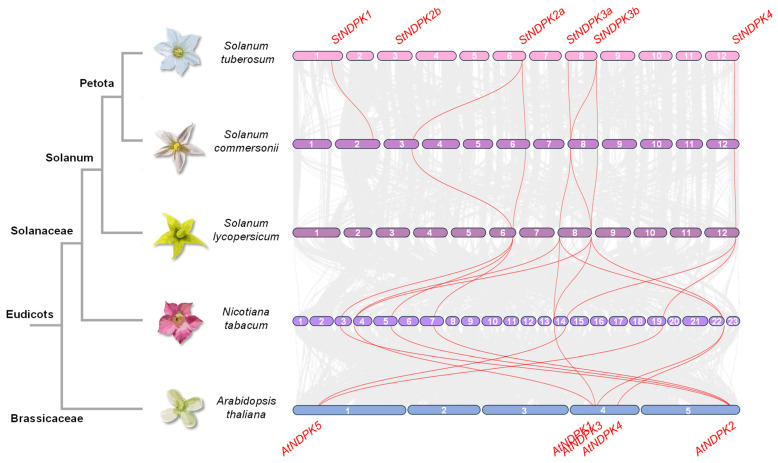
Chromosomal positions and inter-chromosomal groups of *NDPK* gene ortholog pairs in *S. tuberosum*, *S. commersonii*, *S. lycopersicum*, *N. tabacum*, and *A. thaliana*. The chromosomes numbers are indicated. Gray lines in the background demonstrate all syntenic blocks and the red lines exhibit the orthologous genes.

The collinearity between the potato, wild potato, tomato, tobacco, and arabidopsis genomes was compared. Synteny analysis demonstrated high collinearity among potato, wild potato, tomato, tobacco, and arabidopsis, which includes the NDPK genes (Figure 4). Both paralogous and orthologous gene pairs are highlighted in red, and the orthologous pairs are listed in Supplementary Table S3. As expected, the tobacco genome exhibited more extensive duplications once there was a whole-genome duplication event specific to the *Nicotiana* genus [30].

### 2.4. Molecular Dating of NDPK Family Duplication Events in Plants

**Figure 5:**
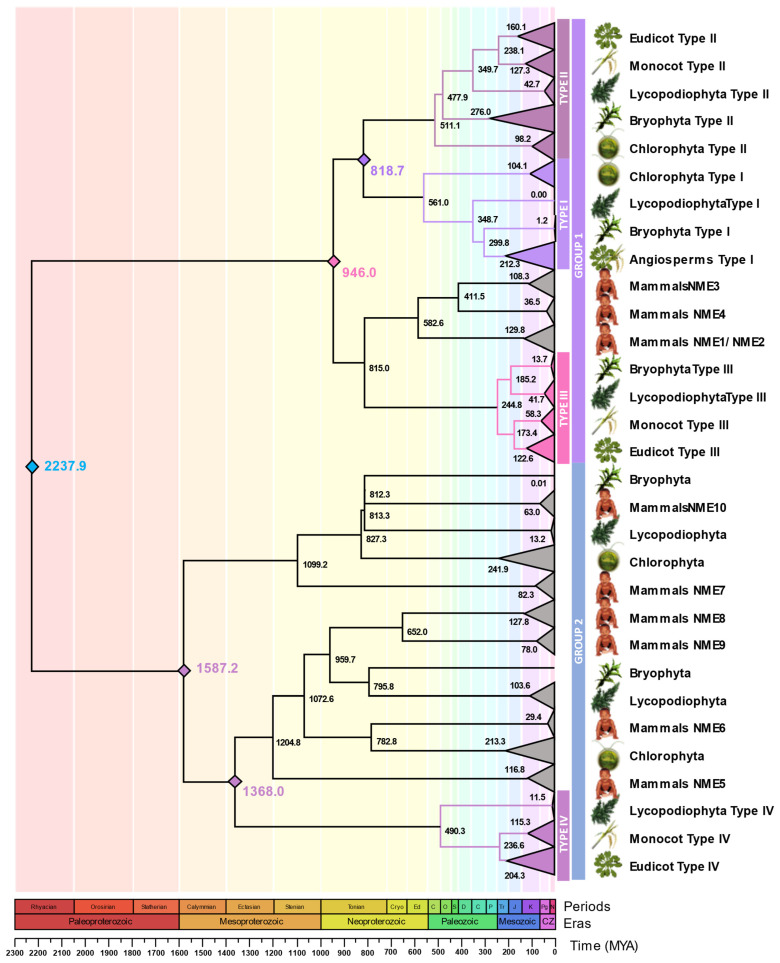
Time-calibrated phylogenetic tree of the NDPK gene family. The tree represents a hypothetical reconstruction of the evolutionary history of NDPK isoforms with estimated divergence times. The main gene duplication events associated with the expansion of the NDPK family are marked by colored diamonds. The different types of plants NDPK are indicated by individual colors. Geological periods are abbreviated as follows: Cryo, Cryogenian; Ed, Ediacaran; Ca, Cambrian; O, Ordovician; S, Silurian; D, Devonian; C, Carboniferous; P, Permian; Tr, Triassic; J, Jurassic; K, Cretaceous; Pg, Paleogene; N, Neogene; Cz, Cenozoic; MYA, million years ago.

*NDPK* genes are present in all living cells, including plants and mammals, where they are known as *NME*. To clarify the evolutionary history and relationships among these isoforms, we investigated the time of their emergence in plants and mammals. Phylogenetic analysis of NDPK sequences suggests that a very ancient duplication event at the onset of the eukaryotic clade led to the formation of group 1 and group 2 isoforms. This event is estimated to have occurred early in the Calymmian period, approximately 1438.4 million years ago (MYA), in a eukaryotic ancestor before the divergence of unikonta and bikonta lineages. This divergence marks the first radiation of eukaryotes, shortly after the emergence of the Last Eukaryotic Common Ancestor (LECA) (Figure 5). Subsequent duplication events contributed to the emergence of different NDPK isoforms in various eukaryotic lineages. Within group 1, the NDPK type I/II and III isoforms are believed to have originated from a duplication event that occurred around 496.5 MYA (Figure 5).

### 2.5. The Expression Pattern of StNDPK Genes in Tuber Development

**Figure 6:**
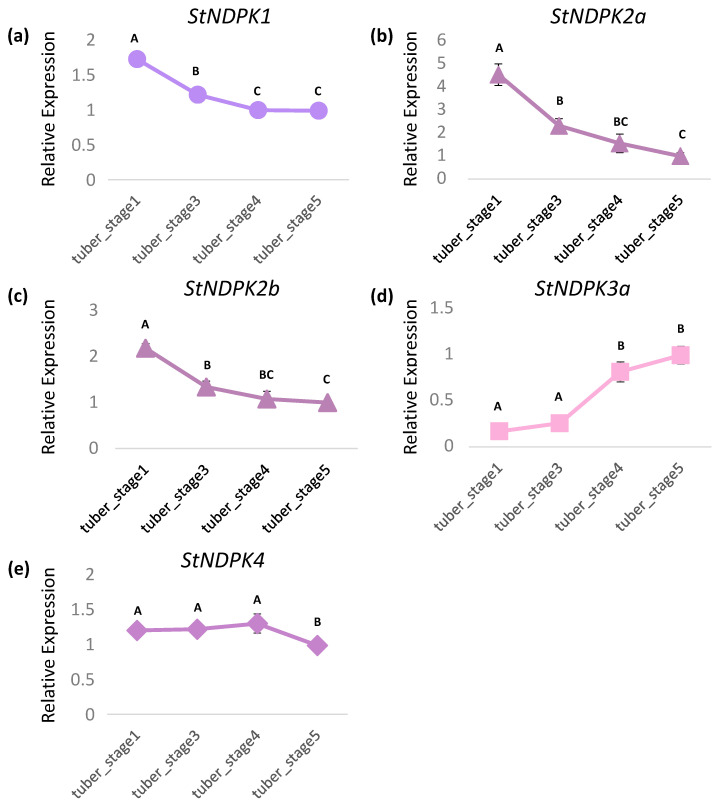
Expression profile of *StNDPK* genes in different stages of tuber development. Relative expression of (**a**) *StNDPK1*, (**b**) *StNDPK2a*, (**c**) *StNDPK2b*, (**d**) *StNDPK3a*, and (**e**) *StNDPK4* genes. The values represent the median among three independent RNA-seq experiments, bars represent standard error, and letters represent statistical difference between samples according to Tukey’s test (*p* < 0.05). The available RNA-seq data did not allow us to reliably determine the expression levels of StNDPK3b. Data is presented as the mean, considering stage 5 as the reference.

Considering the possible role of NDPK during starch accumulation, we decided to explore the expression profile in the potato tuber development. The transcriptome data was accessed in the NCBI (BioProject PRJNA753086) database. *StNDPK1* expression was progressively repressed during tuber development, reaching approximately half of its initial level by stage 5 (Figure 6). Notably, NDPK1 activity has previously been associated with the inhibition of starch formation [22]. Both *StNDPK2a* and *StNDPK2b* genes followed a pattern similar to that observed for *StNDPK1*, with *StNDPK2a* transcript levels declining to approximately 10% of the initial expression, while *StNDPK2b* showed a reduction of roughly 50%. Among type III NDPKs in potato, only *StNDPK3a* showed detectable expression, and it was the sole isoform up-regulated during tuber development, reaching approximately a two-fold increase relative to its initial transcript level. The analysis of the type IV gene *StNDPK4* indicates a modest repression during tuber development, with transcript levels reduced by approximately 10% at stage 5.

### 2.6. Mitochondrial NDPK Activity Increases the ADP Supply to Oxidative Phosphorylation

Mitochondria are the main site of reactive oxygen species (ROS) generation in heterotrophic tissues, and the availability of ADP to oxidative phosphorylation stimulates respiration while concomitantly decreasing ROS production [31,32,33]. The phosphorylation of NDP by NDPK results in the generation of ADP. Thus, we evaluated whether mitochondrial NDPK activity can enhance oxygen consumption and inhibit ROS production through an ADP recycling mechanism.

**Figure 7:**
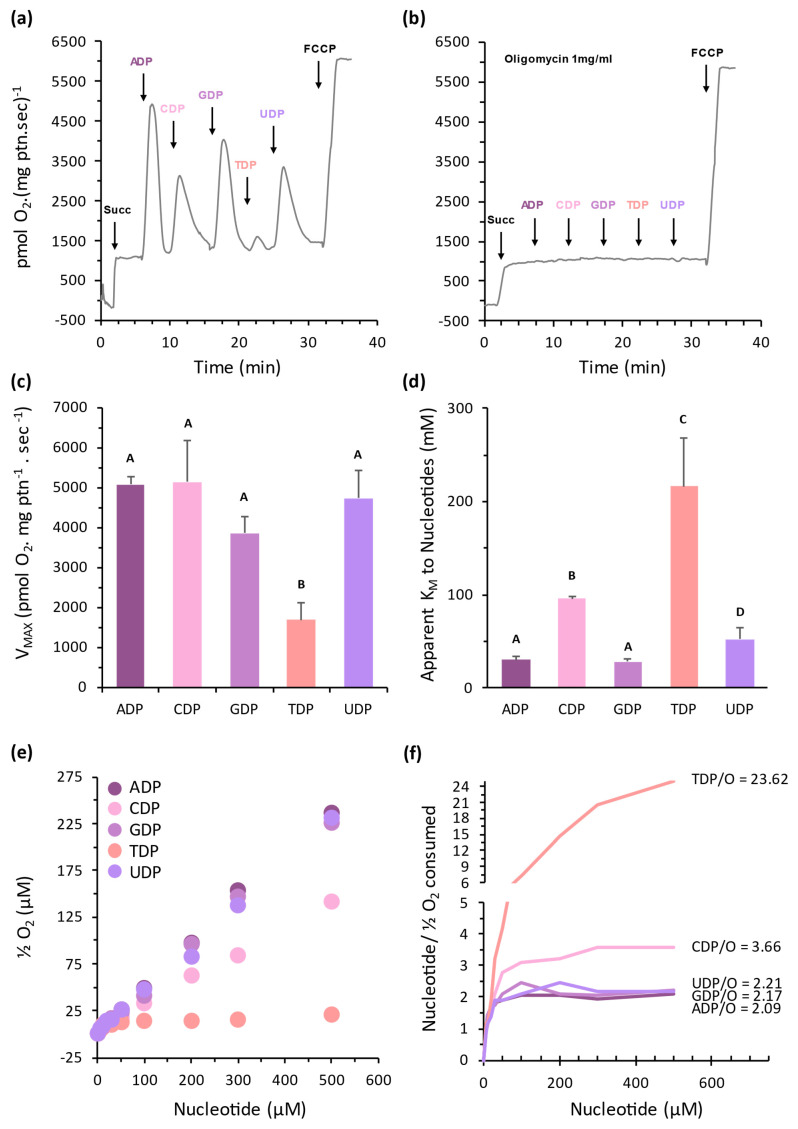
Mitochondrial NDPK activity induces mitochondrial oxygen consumption by the ADP recycling mechanism. (**a**) Oxygen consumption in isolated mitochondria was initiated with 10 mM succinate, followed by the addition of 0.3 mM of each NDP. Maximal oxygen consumption was determined in the presence of 1 mM FCCP. (**b**) Oxygen consumption was also evaluated in the presence of 1 μg/mL oligomycin. (**c**,**d**) Apparent V_MAX_ and K_M_ of isolated mitochondria for different NDPs measured in the presence of 10 mM succinate and 1 mM ATP. Values represent means ± SE of five independent experiments. Different letters above the bars indicate statistically significant differences. (**e**) Correlation between nucleotide concentration and equivalent atomic oxygen consumed in isolated mitochondria in a phosphorylative state. Values represent means ± SE of five independent experiments. (**f**) Ratio between the NDP added and the number of oxygen atoms consumed by mitochondria in a phosphorylative state sustained by 10 mM succinate.

As expected, in isolated potato tuber mitochondria, the addition of 0.1 mM ADP induces the phosphorylative state of respiration, leading to an increase in the oxygen consumption rate. Once ADP is completely converted to ATP through oxidative phosphorylation, the oxygen consumption rate returns to the basal level. Subsequent addition of different NDPs (CDP, GDP, TDP, and UDP) elicits a similar effect, indicating that NDPK phosphorylates NDPs using ATP produced by oxidative phosphorylation, thereby generating ADP and re-inducing the mitochondrial phosphorylative state (Figure 7a). Interestingly, although all tested NDPs were able to induce the phosphorylative state, both the level and amplitude of respiratory activation varied among them, indicating that NDPK exhibits different efficiencies in phosphorylating distinct substrates. At the end of the assays, maximal respiration was assessed by the addition of 1 mM FCCP, an ionophore that uncouples oxygen consumption from ATP synthesis. The addition of 1 μg/mL oligomycin, an inhibitor of F_o_F_1_ ATP synthase, prevented the effect of ADP and other NDPs in respiratory flux, confirming that all substrates stimulated oxygen consumption due to the increase in ADP supply to oxidative phosphorylation (Figure 7b). Importantly, these results refer to mitochondria isolated from whole tuber tissue and do not imply uniform mitochondrial properties across different cell types within the tuber.

To verify the catalytic properties of potato tuber mitochondria regarding the phosphorylation ADP, CDP, GDP, TDP, and UDP, we measured the increase in oxygen consumption induced by crescent concentrations of these substrates. Our results demonstrate that the CDP, GDP, and UDP induced a similar V_MAX_ to ADP, indicating that these NDPs were phosphorylated at the same rate of oxidative phosphorylation. However, the V_MAX_ to TDP corresponded to approximately one-third of V_MAX_ to ADP, indicating that mt-NDPK had a lower ability to phosphorylate TDP, and, consequently, there was a lower supply of ADP to stimulate the mitochondrial oxygen consumption (Figure 7c). In addition, the apparent K_M_ to GDP and UDP was approximately the same as ADP (25 mM), while the apparent K_M_ to CDP and TDP corresponded to about four and eight times this value, respectively (Figure 7d). These data indicate that among the different NDPs, GDP and UDP are preferentially phosphorylated by mt-NDPK at a similar rate of ADP phosphorylation by oxidative phosphorylation.

### 2.7. Stoichiometry Between NDPs Phosphorylated by Mitochondrial Oxygen Consumption

An important parameter to determine the contribution of oxidative phosphorylation in oxygen consumption is the ADP:O ratio, which is the stoichiometry between the number of molecules of ADP phosphorylated per atom of oxygen consumed by mitochondria. The ADP:O directly reflects the number of protons pumped into the IMS and the electron flow by the mitochondrial electron transporter chain. Thus, in intact mitochondria, when succinate, a substrate of complex II, is the source of electrons to ETC, the ADP:O is close to 2.0, because electrons enter the ETC downstream of complex I, bypassing proton translocation at this site and resulting in a lower proton motive force available for ATP synthesis.

To evaluate the contribution of each nucleotide to the rate of oxidative phosphorylation, we analyzed the correlation between nucleotide concentration and the equivalent atomic oxygen consumed in isolated mitochondria (Figure 7e). As expected, GDP and UDP induced oxygen consumption rates comparable to ADP, since they are phosphorylated by NDPK at rates similar to ADP phosphorylation through oxidative phosphorylation. In contrast, the capacity of CDP and TDP to stimulate oxygen consumption was limited. Among them, TDP reached maximal respiratory induction at 50 μM, indicating that this is the upper limit of its phosphorylation by NDPK.

As expected for succinate-dependent oxygen consumption, the ratio between the ADP added and the number of oxygen atoms consumed by mitochondria in the phosphorylative state (ADP:O) was close to 2.0 (Figure 7f). The GDP:O and UDP:O ratios were also close to 2.0, indicating that these nucleotides were fully phosphorylated by mitochondria, generating one equivalent of ADP in IMS. However, the CDP:O and TDP:O ratios were equal to 3.66 and 23.62, respectively, indicating that these nucleotides were not fully phosphorylated in plant mitochondria (Figure 7f).

### 2.8. Mitochondrial NDPK Activity Modulates the Δψ_m_ and ROS Production

**Figure 8:**
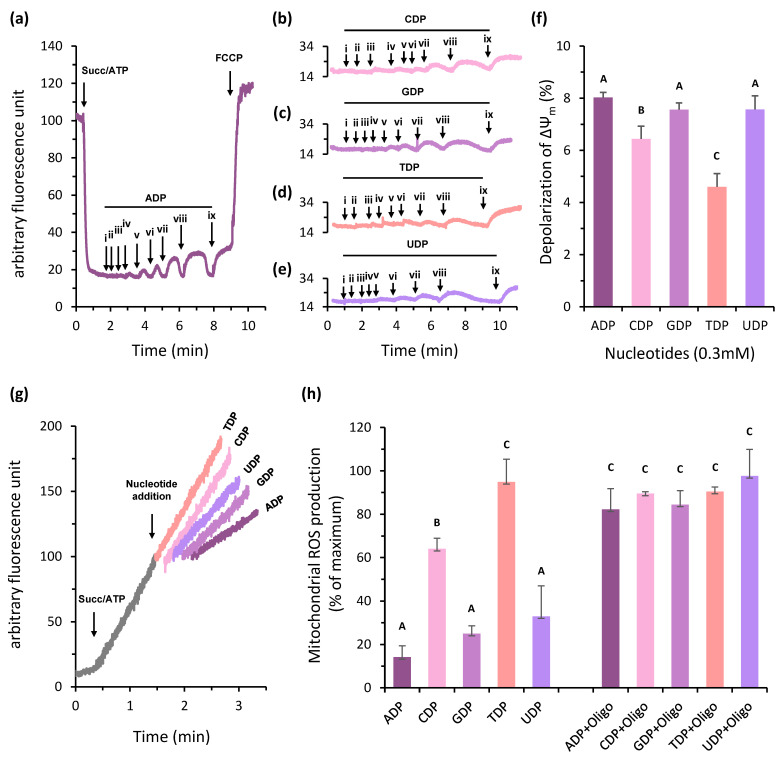
The Δψ_m_ and mitochondrial ROS production are modulated by NDPK activity in plant tuber mitochondria. Δψ_m_ was measured in potato tuber mitochondria, with fluorescence quenching of safranine O. The vertical arrows indicate the addition of ADP (**a**), CDP (**b**), GDP (**c**), TDP (**d**), and UDP (**e**) at the final concentrations of 0.005 mM (i), 0.01 mM (ii), 0.02 mM (iii), 0.03 mM (iv), 0.04 mM (v), 0.05 mM (vi), 0.1 mM (vii), 0.2 mM (viii), and 0.3 mM (ix). The Δψ_m_ was evaluated in the presence of 10 mM succinate (succ) and 1 mM ATP. The Δψ_m_ was totally dissipated by 1 μM FCCP. The figures show a representative experiment. (**f**) Quantification of maximal Δψ_m_ depolarization induced by ADP and other NDPs. The values represent the mean ± SE of five independent experiments and the different letters above the bars indicate statistically significant differences. (**g**) Effect of different NDPs on mitochondrial ROS production. The H_2_O_2_ release was detected by Ampliflu™ Red oxidation after induction of respiration by 10 mM succinate and 1 mM ATP. The nucleotide addition is indicated by a vertical arrow. The figure shows a representative experiment. (**h**) Quantification of H_2_O_2_ release rate in the presence of 0.1 mM of each NDP in the absence and presence of 1 μg/mL oligomycin (Oligo). The values represent the mean ± SE of five independent experiments and the different letters above the bars indicate statistically significant differences.

Previous work has shown that ADP stimulates mitochondrial oxygen consumption by causing a slight dissipation of the Δψ_m_ due to ATP synthase activity [34]. Thus, we verified the ability of mitochondrial NDPK activity in modulating the Δψm in plant mitochondria by ADP production. The Δψm was assessed in the presence of 10 mM succinate and 1 mM ATP, using crescent concentrations of different NDPs. The addition of these substrates was able to reduce the Δψ_m_, and the time of this effect was proportional to the concentration of nucleotide added. In all experiments, we used 1 mM FCCP to fully dissipate the Δψ_m_ (Figure 8a–e). The maximal Δψ_m_ depolarization was determined for each NDP (Figure 8f). ADP induced a maximal depolarization of approximately 8%, with GDP and UDP eliciting comparable effects, reflecting efficient phosphorylation by mitochondrial NDPK. In contrast, CDP and TDP displayed a more limited capacity to depolarize Δψ_m_, reaching maximal values of 6.43% and 4.60%, respectively, suggesting differential substrate specificity of NDPK toward these nucleotides.

As previously described, mitochondrial ROS production is highly dependent on Δψm [34]. Here, we show that mitochondrial NDPK activity can suppress ROS production through the phosphorylation of GDP or UDP in a manner comparable to direct ADP addition, while CDP appears to be less effective. In contrast, TDP failed to inhibit mitochondrial ROS production (Figure 8g,h), consistent with its lower phosphorylation by NDPK in plant mitochondria.

The ability of mitochondrial NDPK to modulate ROS production was further quantified (Figure 8h). The direct addition of ADP to oxidative phosphorylation reduced the mitochondrial ROS production by about 86%, and the activation of mitochondrial NDPK by GDP, UDP, or CDP reduced ROS production by approximately 75%, 70%, and 60%, respectively. As expected, TDP had no detectable antioxidant effect. Notably, the ROS-suppressing effect of all NDPs was abolished by oligomycin, confirming that the reduction of ROS production depends on ATP synthesis within the mitochondrial matrix.

## 3. Discussion

To gain insight into the evolutionary organization of the NDPK gene family in Solanaceae, we performed a comprehensive genome-wide identification and phylogenetic analysis of NDPK genes in different species. Our analysis identified six NDPK genes in potato and tomato, but only three in wild potato and tomato. The genes identified are distributed among the four plant-specific NDPK clades (types I to IV), which are grouped into group 1 (types I, II, and III) and group 2 (type IV). This distribution aligns with previous observations in arabidopsis and rice NDPKs [5]. Furthermore, our analysis suggests that this pattern may be conserved across plants, as we also identified algal type I and type II NDPK genes in group 2, although these do not form a monophyletic clade with plant NDPK IV (Figure 1a and Figure 5). A similar pattern was observed in moss, where NDPK genes were found in all plant-specific clades except type IV (Figure 1a and Figure 5). This absence suggests that type IV NDPK either emerged in the common ancestor of *S. moellendorffii* and angiosperms or was lost in moss.

With origins in bacteria, NDPKs underwent significant diversification in eukaryotes. Our data suggest that the first major duplication, giving rise to group 1 and group 2, occurred shortly after the emergence of LECA (Figure 5). Subsequently, the ancestral lineage diverged around 946 million years ago (MYA) in the common ancestor of plants and animals, separating the progenitor of plant types I and II from type III. Among them, type III NDPK is the orthologous mitochondrial NDPK in animals. The duplication that specifically generated plant types I and II then occurred later, during the early emergence of plants in the Neoproterozoic era. The NDPK domain is conserved among Solanaceae species, rice, and arabidopsis (Figure 1b and Figure 2). In potatoes, all the important residues and sites for NDPK activity in primary protein sequences are conserved, suggesting that these genes can have a biological role preserved.

Type I NDPK has been reported to be important in sugar metabolism, cell wall synthesis, and starch formation [21]. The correlation between NDPK1 silencing and the induction of starch synthesis, as well as its overexpression and the repression of starch synthesis reported in potato, is related to the control of ADP–glucose pyrophosphorylase (AGPase) activity by redox metabolism [17,22]. The analysis of StNDPK1 expression indicates that it is repressed during tuber formation. This pattern is likely important for proper starch formation and tuberization since AGPase is central in this role [35]. StNDPK2a and b follow the same pattern observed for StNDPK1 (Figure 6). This could be linked to NDPK1, whereby less NDPK activity in the cell should promote starch formation [22]. On the other hand, the expression of StNDPK3, the putative mitochondrial isoform, is increased during tuber formation. This may be to compensate for the lower activity in the cytosol and the need for nucleotides for sugar synthesis signaling, as well as for ADP to ATP synthase in the mitochondrial electron transport chain for proper respiration [34]. Together, these patterns suggest a coordinated regulation of NDPK isoforms during tuber development, in which reduced cytosolic NDPK expression may favor starch accumulation, while mitochondrial NDPK activity supports the energetic demands associated with tuberization.

The mitochondrial isoform seems to be present in all plants, and there is a common ancestor, NDPK, with animal mit-NDPK. Indeed, many similarities, such as mitochondrial localization and interaction with AGPase, have been observed between plant type III NDPKs and mitochondrial animal NDPK, where this interaction is important in controlling metastasis suppression [10,18,35].

In plant mitochondria, NDPK activity can induce the phosphorylative state. The addition of different nucleoside diphosphates (NDPs) transiently stimulates oxygen consumption in a manner comparable to ADP, indicating that mitochondrial NDPK can rapidly phosphorylate the supplied NDPs. Indeed, previous studies have shown that in plant mitochondria, the rate of NDP phosphorylation in the intermembrane space by NDPK is nearly equivalent to that of oxidative phosphorylation [27].

Classically, mitochondrial metabolic states were defined by Chance and Williams [36], who described five fundamental states. Among them, state 3 (the phosphorylative state) is characterized by the activation of oxidative phosphorylation by ADP, leading to a high respiratory rate. Activation of oxidative phosphorylation is accompanied by a partial dissipation of Δψm, resulting in increased oxygen consumption and reduced mitochondrial ROS production.

The generation and maintenance of the Δψ_m_ is a central component of the chemiosmotic theory of ATP synthase operation [37], and the optimal conditions for ADP and phosphate supply have been analyzed in the context of thermodynamic buffering [38,39]. Accordingly, the presence of auxiliary buffering enzymes, such as mitochondria-associated kinases, provides a stable flux of ADP to ATP synthase. This concept has been extended to include the role of mitochondrial NDPK in plant mitochondria.

Mitochondrial NDPK exhibits differential capacity to phosphorylate distinct NDPs. The maximal phosphorylation rates of CDP, GDP, and UDP by mitochondrial NDPK are comparable to ADP phosphorylation via oxidative phosphorylation, as reflected by their similar induction of oxygen consumption. In contrast, TDP is phosphorylated at a lower rate, and, consequently, the maximal respiratory flux induced by TDP addition reaches only about one-third of that observed for the other nucleotides. Furthermore, although mitochondrial NDPK can phosphorylate various NDPs, it displays distinct affinities for each substrate. Analysis of the apparent affinities of isolated mitochondria revealed lower affinity for TDP and CDP, whereas the affinities for GDP and UDP were nearly equivalent to that for ADP. These results indicate that in plant mitochondria, GDP and UDP are the preferred NDP substrates for NDPK, while the catalytic efficiency toward CDP and TDP phosphorylation is limited.

In fact, CDP and TDP are preferably directed to nucleic acid synthesis, while the energy balance of GDP and UDP is also important in other physiological processes, such as cell signaling processes and synthesis of polysaccharide precursors [40,41]. In addition, in potato tubers, the homeostasis of uridine nucleotides is especially important to starch synthesis, being involved as substrates in the pathway of sucrose degradation via sucrose synthase and UDP-Glc pyrophosphorylase [42]. Thus, the mitochondrial affinity by GDP and UDP should be much larger than CDP and TDP, as already demonstrated previously [17].

Nucleotide turnover by mitochondrial NDPK likely plays a central role in maintaining a continuous supply of ADP for oxidative phosphorylation, which not only sustains mitochondrial energy metabolism but also contributes to antioxidant defense by preventing excessive Δψ_m_ hyperpolarization. The differential effects observed among NDPs suggest that substrate specificity and phosphorylation efficiency are key determinants of this protective mechanism. GDP and UDP, which are efficiently phosphorylated, appear to support a more robust modulation of Δψ_m_ and suppression of ROS, whereas TDP, with lower apparent affinity, shows minimal impact. These observations highlight mitochondrial NDPK as a modulatory hub, linking nucleotide metabolism to the fine-tuning of mitochondrial redox balance and reinforcing its role in mitigating oxidative stress under physiological conditions.

**Figure 9:**
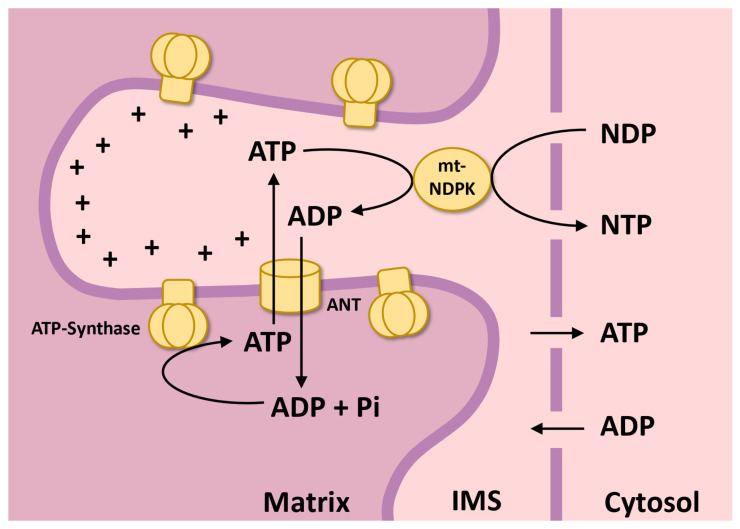
Schematic representation of NDP phosphorylation in the intermembrane space (IMS) by mitochondrial NDPK. In this mechanism, NDPK catalyzes the transfer of a phosphate from ATP to NDP, generating NTP and ADP. ATP is produced in the mitochondrial matrix by ATP synthase using inorganic phosphate (Pi) and is exported to the IMS via the ADP/ATP translocator (ANT). The ADP generated by NDPK can return to the matrix to be re-phosphorylated by ATP synthase, maintaining a constant ADP supply for oxidative phosphorylation. Nucleotides can also equilibrate with the cytosol, linking mitochondrial energy metabolism with cellular nucleotide homeostasis. The “+” sign indicates the accumulation of positive charge, while the arrows indicate solute transport as well as enzymatic reactions.

In addition, we demonstrated that, due to its involvement in general plant nucleotide metabolism, mitochondrial NDPK plays an important role in ADP supply to support oxidative phosphorylation, thereby avoiding the main ATP synthesis-related limitation. Mitochondrial NDPK is able to exert an ADP recycling mechanism in IMS, which contributes to a constant ADP supply to oxidative phosphorylation, resulting in a decrease in the Δψ_m_ and mitochondrial ROS production (Figure 7 and Figure 8). Thus, these data reinforce the idea that mitochondrial ROS production is provided mainly by the equilibration of adenylate levels in mitochondria, and the role of kinases associated with mitochondria appears to be important in this context, exerting an important additional antioxidant defense (Figure 9).

## 4. Materials and Methods

### 4.1. NDPK Gene Identification

Complete NDPK protein sequences of *Volvox carteri*, *Chlamydomonas reinhardtii*, *Selaginella moellendorffii*, *Physcomitrium patens*, sweet potato (*Ipomoea batatas*), tomato (*Solanum lycopersicum*), potato, wild potato (*Solanum commersonii*), wild tomato (*Solanum chilense*), tobacco (*Nicotiana tabacum*), arabica coffee (*Coffea arabica*), common bean (*Phaseolus vulgaris*), and sorghum (*Sorghum bicolor*) were retrieved from NCBI using arabidopsis genes and rice sequences as bait in the protein BLAST (BLASTp) tool (https://blast.ncbi.nlm.nih.gov/Blast.cgi, accessed on 9 March 2026). The sequences obtained from BLAST were used to search protein domains using the Pfam database (https://www.ebi.ac.uk/interpro/entry/pfam/#table, accessed on 9 March 2026). After confirmation of the NDPK domain, the final gene set used for phylogenetic analysis was reported (see Supplementary Table S1); the positions of the NDPK domains for each gene are reported in Supplementary Table S2.

### 4.2. Phylogenetic Tree Reconstruction and Duplication Analysis

The protein sequences from *V. carteri*, *C. reinhardtii*, *S. moellendorffii*, *P. patens*, *I. batatas*, *S. lycopersicum*, *S. tuberosum*, *S. commersonii*, *S. chilense*, *N. tabacum*, *C. arabica*, *A. thaliana*, *P. vulgaris*, *O. sativa*, *S. bicolor*, mice (*Mus musculus*), human (*Homo sapiens*), and *Escherichia coli* were aligned using the Multiple Sequence Comparison by Log Expectation (MUSCLE) tool [43] in Molecular Evolution and Genetic Analysis 12 software (MEGA12) [44] and the gaps were trimmed manually. The maximum likelihood (ML) phylogenetic reconstruction was done using iQTREE2 [45], with 1000 replicates of bootstrap and approximate likelihood ratio test (aLRT) statistic tests. The tree was rooted with *E. coli*. The species tree obtained from TimeTree5 [46] and the NPDK gene tree were used to find gene duplication in MEGA12. The gene duplication tree was used to compute the divergence time using the RealTime-ML tool in MEGA12. All calibrated nodes were inferred by speciation events. The calibration points were provided as minimum and maximum constraints in million years ago: Eukaryote (800–1700), Metazoa (550.25–833), Plantae (469–1891), Embryophyta (469–515.5), (Bryophyta 330.7–515.5), Lycopodiophyta (392.1–451), Angiosperms (125–247.2), Poaceae (65–67), Eudicot (119.6–128.63), Brassicaceae (23.03–23.03), Solanaceae (72–74.3).

### 4.3. Chromosomal Gene Position, Synteny, and Collinearity

The chromosomal gene location in the *S. tuberosum* (Phytozome13, v6.1), *S. lycopersicum* (NCBI, GCF_036512215.1), and *S. commensonii* (NCBI, GCA_018258275.1) genomes was done using Circos Advanced (accessed on 9 March 2026) [47]. Detection of putative gene duplication events was done with MCScanX (2012) (E-value 1 × 10^−10^) in each genome and visualized using TBtools software v1.098769 [48]. Tandem duplication events were defined as two or more homologous genes located on a chromosomal region within 200 kb [49]. Collinearity among *S. teberosum* vs. *S. commersonii* vs. *S. lycopersicum* vs. *N. tabacum* (NCBI, GCA_000715075.2) vs. *A. thaliana* (NCBI, GCF_000001735.4) genomes was done with MCScanX (E-value 1 × 10^−10^) and visualized by TBtools v1.098769 [48].

### 4.4. Protein Primary Structure Analysis

The amino acid sequences of potato, tomato, and arabidopsis were aligned by MUSCLE tool [43] in MEGA 11 software [44] and visualized using ESPript 3.0 (https://espript.ibcp.fr/ESPript/ESPript/, accessed on 9 March 2026) with the similarity-based coloring scheme of MultAlin (accessed on 9 March 2026) at a global score threshold of 0.7 [50]. Motif analyses were performed using MEME v5.5.5 (Multiple EM for Motif Elicitation) (http://meme-suite.org/, accessed on 3 April 2026) for all the species cited above, including wild tomato and rice, with the parameters set to identify 1–10 motifs of width 5–50 [51]. The TargetP-2.0 server was used to predict the presence of N-terminal presequences: signal peptide, mitochondrial transit peptide, chloroplast transit peptide, or thylakoid luminal transit peptide.

### 4.5. RNA-Seq Data Analysis

Raw RNA-sequencing data corresponding to potato tuber maturation stages were retrieved from the NCBI Sequence Read Archive (SRA). Accession numbers included were: SRR15401461 (stage 1), SRR15401462 (stage 1), SRR15401463 (stage 1), SRR15401388 (stage 3), SRR15401399 (stage 3), SRR15401410 (stage 3), SRR15401421 (stage 4), SRR15401432 (stage 4), SRR15401443 (stage 4), SRR15401454 (stage 5), SRR15401465 (Stage 5), and SRR15401466 (stage 5). No publicly available RNA-seq data were identified for Stage 2.

SRA files were downloaded using the prefetch utility (v.3.0.3) and subsequently converted to compressed FASTQ format with fastq-dump (v2.11.3), enabling paired-end read separation. Initial quality assessment of the raw sequencing reads was performed using FastQC v0.12.1 (https://github.com/s-andrews/FastQC, last accessed on 19 February 2026). Adapter trimming and removal of low-quality bases were conducted using Trim Galore v0.6.6 (https://github.com/FelixKrueger/TrimGalore, last accessed on 19 February 2026), using cutadapt v4.4 with Python 3.12.3. Paired-end mode was enabled, and a clipping of 5 bp from both read ends was done to eliminate potential sequence bias and low-confidence regions. For alignment, a STAR v2.7.11b [52] genome index was generated using the *S. tuberosum* DM1-3 516 R44 v6.1 genome assembly and its corresponding high-confidence gene model annotation (GFF3 format). Index generation incorporated splice junction information and used an overhang parameter of 44, reflecting the effective read length after trimming. Trimmed paired-end reads were then aligned to the indexed genome with STAR in two-pass mode, producing sorted BAM files directly. Alignment statistics were obtained with samtools 1.22.1 [53], including chromosome-level mapping summaries. Gene-level quantification was performed using featureCounts v2.0.6 [54], specifying paired-end read counting, GFF3 annotation parsing, and exon-based assignment grouped by the parent attribute to obtain transcript-level read counts. Only uniquely mapped, properly paired reads were retained for quantification.

### 4.6. Isolation of Potato Tuber Mitochondria by Self-Generated Percoll Gradient

Potato tuber mitochondria were obtained as previously described [55] with some modifications. Prior to extraction, potato tubers were peeled, and any visible eyes were removed. Total mitochondria were isolated using a cold extraction buffer containing: 10 mM HEPES/Tris pH 7.4, 0.3 M mannitol, 2 mM EGTA, 5 mM EDTA, 0.3 mM phenylmethylsulfonyl fluoride, 20 mM β-mercaptoethanol, and 0.1 g% (*w*/*v*) fatty acid-free bovine serum albumin (faf-BSA). The homogenate was strained through eight layers of cheesecloth and centrifuged at 3000× *g* at 4 °C for 3 min. The supernatant was centrifuged at 12,000× *g* at 4 °C for 10 min. The mitochondrial pellet was resuspended in 5 mL of ice-cold extraction buffer and layered in Hitachi P50 centrifuge tubes containing 35 mL of cold extraction buffer containing 28% (*v*/*v*) Percoll and centrifuged at 40,000× *g* at 4 °C for 30 min. The mitochondrial fraction was removed and diluted with extraction buffer without Percoll and centrifuged twice at 12,000× *g* at 4 °C for 10 min. The final pellet was resuspended in 0.6 mL of extraction buffer and kept in an ice-water bath. The final protein concentration was quantified using the Bradford method [56] and varied from 10 to 20 mg/mL.

### 4.7. Oxygen Consumption Measurements

Oxygen consumption rates were measured polarographically using high-resolution respirometry (Oroboros Oxygraph-O2K). The electrode was calibrated between 0 and 100% saturation with atmospheric oxygen at 28 °C. The isolated mitochondria (0.2 mg/mL) were incubated with 2.0 mL of the standard respiration buffer containing 0.3 M mannitol, 10 mM Tris-HCl pH 7.2, 3 mM MgSO_4_, 10 mM NaCl, 5 mM KH_2_PO_4_, 0.3 mM β-NAD^+^, and 0.1% (*v*/*v*) faf-BSA.

### 4.8. ∆ψ_m_ Determination

The ∆ψ_m_ was measured by using the fluorescence signal of the cationic dye safranine O, which is accumulated and quenched inside energized mitochondria [57]. Isolated mitochondria (0.2 mg protein/mL) were incubated in the standard respiration buffer (see oxygen consumption measurements) supplemented with 15 mM safranin O, and 1 mM FCCP was used as a positive control to collapse ∆ψ_m_. Fluorescence was detected with an excitation wavelength of 495 nm (slit 5 nm) and an emission wavelength of 586 nm (slit 5 nm). Data were reported as arbitrary fluorescence units.

### 4.9. Determination of Mitochondrial H_2_O_2_ Release

The H_2_O_2_ released from potato tuber mitochondria was determined by the Ampliflu™ Red (Sigma Aldrich, St. Louis, MO, USA) oxidation method, as previously described [58]. Briefly, mitochondria (0.2 mg protein/mL) were incubated in the standard respiration buffer (see oxygen consumption measurements) supplemented with 10 mM Ampliflu Red and 5 units/mL horseradish peroxidase. Fluorescence was monitored at excitation and emission wavelengths of 563 nm (slit 5 nm) and 587 nm (slit 5 nm), respectively. Calibration was performed by the addition of known quantities of H_2_O_2_.

### 4.10. Statistical Analysis

Data were plotted with Microsoft Excel (Microsoft Office 365, Microsoft Corp., Redmond, WA, USA) and analyzed by *t*-test using GraphPad Prism 5. *p*-values of 0.05 were considered statistically significant.

## 5. Conclusions

In conclusion, our integrative evolutionary, expression, and functional analyses provide new insights into the diversification and physiological roles of NDPKs in plants. We show that plant NDPK types are highly conserved across lineages, with early duplication events shaping their current diversity, and that Solanum species retain lineage-specific expansions. During potato tuber development, most cytosolic NDPK isoforms are transcriptionally repressed, a pattern consistent with the metabolic shift toward starch accumulation. In contrast, the mitochondrial isoform StNDPK3 is specifically up-regulated, supporting a central role in sustaining nucleotide homeostasis and ADP supply for oxidative phosphorylation. By linking mitochondrial NDPK activity to Δψ_m_ modulation, respiratory efficiency, and suppression of mitochondrial ROS production, our findings highlight mitochondrial NDPK as a key modulatory hub connecting energy metabolism, redox balance, and developmental reprogramming during tuberization.

## Data Availability

All data presented in this study are available upon request from the corresponding author.

## Supplementary Materials

The following supporting information can be downloaded at https://www.mdpi.com/article/10.3390/plants15081156/s1: Table S1: List of genes used in the phylogenetic analysis. The table includes the species, gene ID, and gene name adopted in this study; Table S2: Results of Pfam NDPK (NDK) domain identification. The table includes the Pfam accession code, protein ID, e-value, score, and domain coordinates (start and end positions) for each query sequence; Table S3: Linked gene pairs identified in the collinearity analysis. For each gene pair, the table reports the species name, chromosome, and gene ID of both genes, presented on the same row.
