# Supplementary material for: Nucleoside Diphosphate Kinase Family: Evolutionary Analysis and Protective Role in Mitochondrial ROS Production
Source: Plants (Basel). 2026 Apr 9;15(8):1156. doi: 10.3390/plants15081156 (PMC13120501; doi:10.3390/plants15081156)
Supplement: Supplementary file 1 [file plants-15-01156-s001.zip › plants-4222200-supplementary.pdf]

Supplementary Table S1. List of genes used in the phylogenetic analysis. The table includes the species. gene ID. and the gene name adopted in this study.

Species	Gene ID	Name
Arabidopsis thaliana (At)	AT4G09320.1	AtNPDK1
	AT5G63310.1	AtNPDK2
	AT4G11010.1	AtNPDK3
	AT4G23900.1	AtNPDK4
	AT1G17410.1	AtNPDK5
Solanum tuberosum (St)	Soltu.DM.01G030520.1	StNDPK1
	Soltu.DM.03G024360.1	StNDPK2a
	Soltu.DM.06G027190.1	StNDPK2b
	Soltu.DM.08G029260.1	StNDPK3a
	Soltu.DM.08G004760.1	StNDPK3b
	Soltu.DM.12G009010.1	StNDPK4
Solanum commersoni (Sc)	KAG5617898.1	ScNDPK2a
	KAG5602487.1	ScNDPK2b
	KAG5572977.1	ScNDPK4
Solanum lycopersicum (Sl)	XP_004236354.1-Solyc	SlNDPK2a
	XP_004241524.1-Solyc	SlNDPK2b
	XP_004244635.1-Solyc	SlNDPK3b
	XP_004252406.1-Solyc	SlNDPK4
	NP_001234174.2	SlNDPK1
	XP_010325356.1-Solyc	SlNDPK3a
Solanum chilensis (Schi)	TMW87693.1-Sochi	SchNDPK2a
	TMX04228.1-Sochi	SchNDPK2b
	TMW86799.1-Sochi	SchNDPK34
Nicotiana tabacum (Nt)	NP_001311894.1	NtNPDK1a
	NP_001412804.1	NtNPDK1b
	XP_016443335.1	NtNPDK1c
	XP_016446591.1	NtNPDK1d
	XP_016473098.1	NtNPDK2a
	XP_016481074.1	NtNPDK2b
	XP_016485138.1	NtNPDK2c
	XP_016495271.1	NtNPDK3a
	XP_016497593.1	NtNPDK3b
	XP_016499244.1	NtNPDK3c
	XP_016507937.1	NtNPDK3d
	XP_016512429.1	NtNPDK4a
	XP_075084596.1	NtNPDK4b
	XP_075087247.1	NtNPDK4c
	XP_075096789.1	NtNPDK4d
	XP_075096790.1	NtNPDK4e

Supplementary Table S1. List of genes used in the phylogenetic analysis. The table includes the species. gene ID. and the gene name adopted in this study. (Continued)

Coffea arabica (Ca)	XP_027113400.1	CaNPDK1a
	XP_027106602.1	CaNPDK1b
	XP_071902018.1	CaNPDK2a
	XP_027121423.2	CaNPDK2b
	XP_027081235.1	CaNPDK3a
	XP_027081236.1	CaNPDK3b
	XP_027085156.1	CaNPDK3c
	XP_027125064.1	CaNPDK4a
	XP_071903024.1	CaNPDK4b
	XP_027125232.1	CaNPDK4c
	XP_027125230.1	CaNPDK4d
	XP_027125231.1	CaNPDK4e
Ipoema batatas (Ib)	GMD62480.1	IbNPDK1a
	GMD38032.1	IbNPDK1b
	GMD71649.1	IbNPDK1c
	GMD33560.1	IbNPDK2
	GMD20876.1	IbNPDK4a
	GMC47616.1	IbNPDK4b
	GMC51929.1	IbNPDK4c
	GMC55733.1	IbNPDK4d
Phaseolus vulgaris (Pv)	XP_068478240.1	PvNPDK1
	XP_068464915.1	PvNPDK2
	XP_068471002.1	PvNPDK3
	XP_068479250.1	PvNPDK4
Oryza sativa (Os)	LOC_Os07g30970.1	OsNDPK1
	LOC_Os12g36194.1	OsNDPK2
	LOC_Os05g51700.1	OsNDPK3
	LOC_Os10g41410.1	OsNDPK4
	LOC_Os02g35700.1	OsNDPK5
Sorghum bicolor (Sb)	XP_002462878.1	SbNPDK1
	XP_002442297.1	SbNPDK2
	XP_021303667.1	SbNPDK3
	XP_002464921.1	SbNPDK4
	XP_002452303.2	SbNPDK5
Selaginella moellendorffii (Sm)	XP_002970181.1	SmNPDK1a
	XP_002978348.2	SmNPDK1b
	XP_024535825.1	SmNPDK2a
	XP_002977612.1	SmNPDK2b
	XP_002974524.1	SmNPDK3a
	XP_002963221.1	SmNPDK3b
	XP_002992366.1	SmNPDK4a
	XP_024528465.1	SmNPDK4b
	XP_002960640.2	SmNPDK5a
	XP_024541912.1	SmNPDK5b
	XP_024543453.1	SmNPDK5c
	XP_002961733.1	SmNPDK6a
	XP_024525285.1	SmNPDK6b
	XP_024525283.1	SmNPDK6c
	XP_024525284.1	SmNPDK6d
	XP_024537472.1	SmNPDK6e

Supplementary Table S1. List of genes used in the phylogenetic analysis. The table includes the species. gene ID. and the gene name adopted in this study. (Continued)

Physcomitrella patens (Pp)	XP_024366352.1	PpNPDK6d
	XP_024368945.1	PpNPDK1a
	XP_024368299.1	PpNPDK1b
	XP_024392429.1	PpNPDK2a
	XP_024390257.1	PpNPDK2b
	XP_024370924.1	PpNPDK2c
	XP_024370925.1	PpNPDK2d
	XP_024370926.1	PpNPDK2e
	XP_024398552.1	PpNPDK3a
	XP_024367932.1	PpNPDK3b
	XP_024398553.1	PpNPDK3c
	XP_024366539.1	PpNPDK5
	XP_024366354.1	PpNPDK6a
	XP_073387667.1	PpNPDK6b
	XP_024366355.1	PpNPDK6c
	XP_073387668.1	PpNPDK6e
Chlamydomonas reinhardtii (Cr)	XP_042915431.1	CrNPDK1a
	XP_001698246.1	CrNPDK1b
	XP_001702884.2	CrNPDK2
	XP_001698136.1	CrNPDK5
	XP_001702841.1	CrNPDK6
Volvox carteri (Vc)	XP_002952178.1	VcNPDK1
	XP_002955131.1	VcNPDK2
	XP_002950816.1	VcNPDK5
	XP_002946347.1	VcNPDK6
Escherichia coli (Ec)	P0A763	EcNDPK
Homo sapiens (Hs)	NP_937818	HsNME_1
	NP_001018148	HsNME_2
	NP_002504	HsNME_3
	NP_003542	HsNME_4
	NP_005000	HsNME_5
	NP_005784	HsNME_6
	NP_037462	HsNME_7
	NP_057700	HsNME_8
	NP_835231	HsNME_9
	NP_008846	HsNME_10
Mus musculus (Mm)	NP_032730	MmNME_1
	NP_032731	MmNME_2
	NP_062704	MmNME_3
	NP_062705	MmNME_4
	NP_542368	MmNME_5
	NP_061227	MmNME_6
	NP_612187	MmNME_7
	NP_853622	MmNME_8
	XP_893103	MmNME_9
	NP_598430	MmNME_10

Supplementary Table S2. Results of Pfam NDPK (NDK) domain identification. The table includes the Pfam accession code. protein ID. e-value. score. and domain coordinates (start and end positions) for each query sequence.

	pfam_id	accession	protein_id	evalue	score	start	end
Cr	NDK	PF00334.24	XP_001698136.1-Chre	8E-46	1557	5	139
	NDK	PF00334.24	XP_001698246.1-Chre	4E-55	1858	4	135
	NDK	PF00334.24	XP_001702841.1-Chre	2.8E-25	1410	92	224
	NDK	PF00334.24	XP_001702841.1-Chre	4.4E-18	691	237	374
	NDK	PF00334.24	XP_001702884.2-Chre	1.3E-53	1842	17	147
	NDK	PF00334.24	XP_042915431.1-Chre	4E-55	1858	4	135
Ca	NDK	PF00334.24	XP_027081235.1-Coara	4.4E-54	1857	88	221
	NDK	PF00334.24	XP_027081236.1-Coara	2.1E-53	1835	322	455
	NDK	PF00334.24	XP_027085156.1-Coara	8.5E-53	1815	322	455
	NDK	PF00334.24	XP_027106602.1-Coara	4.6E-56	1921	2	133
	NDK	PF00334.24	XP_027113400.1-Coara	4.6E-56	1921	2	133
	NDK	PF00334.24	XP_027121423.2-Coara	1.6E-55	1904	94	228
	NDK	PF00334.24	XP_027125064.1-Coara	3E-44	1506	39	172
	NDK	PF00334.24	XP_027125230.1-Coara	4.8E-43	1499	37	172
	NDK	PF00334.24	XP_027125231.1-Coara	4.8E-43	1499	37	172
	NDK	PF00334.24	XP_027125232.1-Coara	3.9E-41	1438	1	131
	NDK	PF00334.24	XP_071902018.1-Coara	1.4E-55	1905	94	228
	NDK	PF00334.24	XP_071903024.1-Coara	3E-44	1506	39	172
Nt	NDK	PF00334.24	NP_001311894.1-Nitab	1.1E-54	1876	85	218
	NDK	PF00334.24	NP_001412804.1-Nitab	3.9E-40	1891	2	133
	NDK	PF00334.24	XP_016443335.1-Nitab	6.8E-50	1721	2	128
	NDK	PF00334.24	XP_016446591.1-Nitab	5.1E-55	1887	2	133
	NDK	PF00334.24	XP_016473098.1-Nitab	6.4E-29	1495	37	171
	NDK	PF00334.24	XP_016481074.1-Nitab	6.7E-28	1462	37	170
	NDK	PF00334.24	XP_016485138.1-Nitab	1.1E-53	1844	89	222
	NDK	PF00334.24	XP_016495271.1-Nitab	2.8E-55	1895	2	133
	NDK	PF00334.24	XP_016497593.1-Nitab	5.6E-54	1854	89	222
	NDK	PF00334.24	XP_016499244.1-Nitab	1.2E-38	1843	88	221
	NDK	PF00334.24	XP_016507937.1-Nitab	7.1E-55	1882	78	211
	NDK	PF00334.24	XP_016512429.1-Nitab	3.1E-39	1862	89	222
	NDK	PF00334.24	XP_075084596.1-Nitab	1.2E-38	1843	88	221
	NDK	PF00334.24	XP_075087247.1-Nitab	6.4E-29	1495	37	171
	NDK	PF00334.24	XP_075096789.1-Nitab	2.4E-14	1056	37	133
	NDK	PF00334.24	XP_075096790.1-Nitab	1.4E-14	1064	37	133
Pv	NDK	PF00334.24	XP_068464915.1-Phvul	5.7E-39	1853	81	213
	NDK	PF00334.24	XP_068471002.1-Phvul	1.1E-37	1811	320	453
	NDK	PF00334.24	XP_068478240.1-Phvul	2.9E-56	1928	3	134
	NDK	PF00334.24	XP_068479250.1-Phvul	1.4E-44	1549	46	180

Supplementary Table S2. Results of Pfam NDPK (NDK) domain identification. The table includes the Pfam accession code. protein ID. e-value. score. and domain coordinates (start and end positions) for each query sequence. (Continued)

St	NDK	PF00334.24	Soltu.DM.08G029260.1	1.3E-53	1841	89	222
	NDK	PF00334.24	Soltu.DM.06G027190.1	1.2E-55	1907	77	210
	NDK	PF00334.24	Soltu.DM.01G030520.1	5.3E-55	1887	2	133
	NDK	PF00334.24	Soltu.DM.03G024360.1	8E-55	1848	88	221
	NDK	PF00334.24	Soltu.DM.08G004760.1	7.6E-54	1849	83	216
	NDK	PF00334.24	Soltu.DM.12G009010.1	1.2E-44	1551	37	171
Sm	NDK	PF00334.24	XP_002960640.2-Semoe	8.8E-32	1135	115	249
	NDK	PF00334.24	XP_002961733.1-Semoe	6E-41	1399	92	226
	NDK	PF00334.24	XP_002961733.1-Semoe	7E-19	685	237	373
	NDK	PF00334.24	XP_002963221.1-Semoe	4.2E-53	1825	86	219
	NDK	PF00334.24	XP_002970181.1-Semoe	2.3E-55	1899	5	136
	NDK	PF00334.24	XP_002974524.1-Semoe	4.2E-53	1825	86	219
	NDK	PF00334.24	XP_002977612.1-Semoe	1.7E-54	1870	74	207
	NDK	PF00334.24	XP_002978348.2-Semoa	2.3E-55	1899	5	136
	NDK	PF00334.24	XP_002992366.1-Semoe	2.6E-44	1540	23	156
	NDK	PF00334.24	XP_024525283.1-Semoe	3E-41	1409	92	225
	NDK	PF00334.24	XP_024525283.1-Semoe	3.3E-08	371	271	408
	NDK	PF00334.24	XP_024525284.1-Semoe	2.8E-25	1410	92	225
	NDK	PF00334.24	XP_024525284.1-Semoe	2.5E-12	505	267	391
	NDK	PF00334.24	XP_024525285.1-Semoe	2.8E-25	1410	92	225
	NDK	PF00334.24	XP_024525285.1-Semoe	2.2E-13	539	237	390
	NDK	PF00334.24	XP_024528465.1-Semoe	3.8E-45	1568	23	156
	NDK	PF00334.24	XP_024535825.1-Semoe	1.8E-39	1869	74	207
	NDK	PF00334.24	XP_024537472.1-Semoe	6.6E-26	1398	92	226
	NDK	PF00334.24	XP_024537472.1-Semoe	2E-13	508	268	391
	NDK	PF00334.24	XP_024541912.1-Semoe	2E-16	1123	120	254
	NDK	PF00334.24	XP_024543453.1-Semoe	2.1E-16	1123	126	260
Sl	NDK	PF00334.24	NP_001234174.2-Solyc	1.6E-55	1903	2	133
	NDK	PF00334.24	XP_004236354.1-Solyc	1.4E-54	1873	86	219
	NDK	PF00334.24	XP_004241524.1-Solyc	4.6E-55	1889	77	210
	NDK	PF00334.24	XP_004244635.1-Solyc	7.8E-40	1849	85	218
	NDK	PF00334.24	XP_004252406.1-Solyc	4.8E-30	1564	40	174
	NDK	PF00334.24	XP_010325356.1-Solyc	1.3E-53	1841	89	222
Sc	NDK	PF00334.24	KAG5572977.1	1.4E-44	1549	26	160
	NDK	PF00334.24	KAG5617898.1	1.4E-54	1873	85	218
	NDK	PF00334.24	KAG5602487.1	2.7E-39	1864	77	210

Supplementary Table S2. Results of Pfam NDPK (NDK) domain identification. The table includes the Pfam accession code. protein ID. e-value. score. and domain coordinates (start and end positions) for each query sequence. (Continued)

Sch	NDK	PF00334.24	TMW86799.1-Sochi	6E-46	1561	40	174
	NDK	PF00334.24	TMX04228.1-Sochi	2.7E-39	1863	86	219
	NDK	PF00334.24	TMW87693.1-Sochi	3.3E-55	1893	77	210
Sb	NDK	PF00334.24	XP_002442297.1-Sobic	1E-39	1845	75	208
	NDK	PF00334.24	XP_002452303.2-Sobic	1.2E-44	1552	92	227
	NDK	PF00334.24	XP_002462878.1-Sobic	1.9E-40	1901	3	134
	NDK	PF00334.24	XP_002464921.1-Sobic	2.9E-56	1928	2	133
	NDK	PF00334.24	XP_021303667.1-Sobic	2.8E-53	1831	94	227
Pp	NDK	PF00334.24	XP_024366352.1-Phpat	4E-41	1405	95	226
	NDK	PF00334.24	XP_024366352.1-Phpat	2.4E-17	667	239	377
	NDK	PF00334.24	XP_024366354.1-Phpat	4E-41	1405	95	226
	NDK	PF00334.24	XP_024366354.1-Phpat	2.4E-17	667	239	377
	NDK	PF00334.24	XP_024366355.1-Phpat	4E-41	1405	95	226
	NDK	PF00334.24	XP_024366355.1-Phpat	2.4E-17	667	239	377
	NDK	PF00334.24	XP_024366539.1-Phpat	1.6E-34	1223	13	145
	NDK	PF00334.24	XP_024367932.1-Phpat	1.1E-51	1780	86	219
	NDK	PF00334.24	XP_024368299.1-Phpat	4.4E-54	1857	5	136
	NDK	PF00334.24	XP_024368945.1-Phpat	4.4E-54	1857	5	136
	NDK	PF00334.24	XP_024370924.1-Phpat	2.2E-17	669	106	170
	NDK	PF00334.24	XP_024370925.1-Phpat	7.4E-18	684	43	107
	NDK	PF00334.24	XP_024370926.1-Phpat	5.5E-18	688	32	96
	NDK	PF00334.24	XP_024390257.1-Phpat	3.6E-51	1762	69	202
	NDK	PF00334.24	XP_024392429.1-Phpat	1.4E-51	1776	55	188
	NDK	PF00334.24	XP_024398552.1-Phpat	1.1E-51	1780	86	219
	NDK	PF00334.24	XP_024398553.1-Phpat	2.9E-52	1798	7	140
	NDK	PF00334.24	XP_073387667.1-Phpat	4E-41	1405	95	226
	NDK	PF00334.24	XP_073387667.1-Phpat	2.4E-17	667	239	377
	NDK	PF00334.24	XP_073387668.1-Phpat	4E-41	1405	95	226
	NDK	PF00334.24	XP_073387668.1-Phpat	2.4E-17	667	239	377

Supplementary Table S3. Linked gene pairs identified in the collinearity analysis. For each gene pair. the table reports the species name. chromosome. and gene ID of both genes. presented on the same row.

S. tuberosum	Chromossome	Gene ID		S. comersoni	Chromossome	Gene ID
StNDPK1	ST4.03ch01	PGSC0003DMT400080042.v4.03		-	CM031107.1	rna-SC_LZ32_Chr02_76010T
StNDPK2b	ST4.03ch06	PGSC0003DMT400069352.v4.03		ScNDPK2a	CM031108.1	rna-SC_LZ32_Chr03_42210T
StNDPK2b	ST4.03ch06	PGSC0003DMT400069352.v4.03		ScNDPK2b	CM031111.1	rna-SC_LZ32_Chr06_43400T
StNDPK3a	ST4.03ch08	PGSC0003DMT400031997.v4.03	Linked to	-	CM031113.1	rna-SC_LZ32_Chr08_45360T
StNDPK3b	ST4.03ch08	PGSC0003DMT400014828.v4.03		-	CM031113.1	rna-SC_LZ32_Chr08_05900T
StNDPK3a	ST4.03ch08	PGSC0003DMT400031997.v4.03		-	CM031113.1	rna-SC_LZ32_Chr08_05900T
StNDPK4	ST4.03ch12	PGSC0003DMT400011148.v4.03		ScNDPK4	CM031117.1	rna-SC_LZ32_Chr12_39370T

S. comersoni	Chromossome	Gene ID		S. lycopersicum	Chromossome	Gene ID
ScNDPK2a	CM031108.1	rna-SC_LZ32_Chr03_42210T		SlNDPK2b	NC_090805.1	rna-XM_004241476.4
ScNDPK2b	CM031111.1	rna-SC_LZ32_Chr06_43400T		SlNDPK2b	NC_090805.1	rna-XM_004241476.4
-	CM031113.1	rna-SC_LZ32_Chr08_05900T	Linked to	SlNDPK3b	NC_090807.1	rna-XM_004244587.5
-	CM031113.1	rna-SC_LZ32_Chr08_45360T		SlNDPK3a	NC_090807.1	rna-XM_010327054.4
-	CM031113.1	rna-SC_LZ32_Chr08_05880T		SlNDPK3a	NC_090807.1	rna-XM_010327054.4
ScNDPK4	CM031117.1	rna-SC_LZ32_Chr12_39370T		SlNDPK4	NC_090811.1	rna-XM_004252358.5

S. lycopersicum	Chromossome	Gene ID		N. tabacum	Chromossome	Gene ID
SlNDPK2b	NC_090805.1	rna-XM_004241476.4		NtNPDK1a	NC_134083.1	rna-NM_001324965.1
SlNDPK2b	NC_090805.1	rna-XM_004241476.4		-	NC_134085.1	rna-XM_016656943.2
SlNDPK2b	NC_090805.1	rna-XM_004241476.4		NtNPDK3d	NC_134087.1	rna-XM_016652451.2
SlNDPK3b	NC_090807.1	rna-XM_004244587.5		NtNPDK3b	NC_134084.1	rna-XM_016642107.1
SlNDPK3a	NC_090807.1	rna-XM_010327054.4		NtNPDK3b	NC_134084.1	rna-XM_016642107.1
SlNDPK3b	NC_090807.1	rna-XM_004244587.5	Linked to	-	NC_134094.1	rna-XM_016629652.2
SlNDPK3a	NC_090807.1	rna-XM_010327054.4		-	NC_134094.1	rna-XM_016629652.2
SlNDPK3a	NC_090807.1	rna-XM_010327054.4		NtNPDK3c	NC_134102.1	rna-XM_016643758.2
SlNDPK3b	NC_090807.1	rna-XM_004244587.5		NtNPDK3c	NC_134102.1	rna-XM_016643758.2
SlNDPK4	NC_090811.1	rna-XM_004252358.5		NtNPDK2a	NC_134094.1	rna-XM_016617612.2
SlNDPK4	NC_090811.1	rna-XM_004252358.5		NtNPDK4d	NC_134099.1	rna-XM_075240688.1

N.tabacum	Chromossome	Gene ID		A. thaliana	Chromossome	Gene ID
NtNPDK1a	NC_134083.1	rna-NM_001324965.1		AtNPDK2	NC_003076.8	rna-NM_125726.4
NtNPDK3b	NC_134084.1	rna-XM_016642107.1		AtNPDK3	NC_003075.7	rna-NM_117171.4
-	NC_134085.1	rna-XM_016656943.2		AtNPDK2	NC_003076.8	rna-NM_125726.4
NtNPDK3d	NC_134087.1	rna-XM_016652451.2		AtNPDK2	NC_003076.8	rna-NM_125726.4
NtNPDK4a	NC_134094.1	rna-XM_016617612.2	Linked to	AtNDPK5	NC_003070.9	rna-NM_101602.4
-	NC_134094.1	rna-XM_016629652.2		AtNPDK3	NC_003075.7	rna-NM_117171.4
NtNPDK4d	NC_134099.1	rna-XM_075240688.1		AtNDPK5	NC_003070.9	rna-NM_101602.4
-	NC_134102.1	rna-XM_016643758.2		AtNPDK4	NC_003075.7	rna-NM_118522.3
-	NC_134102.1	rna-XM_016643758.2		AtNPDK3	NC_003075.7	rna-NM_117171.4
